# Linear Dispersion Relation and Depth Sensitivity to Swell Parameters: Application to Synthetic Aperture Radar Imaging and Bathymetry

**DOI:** 10.1155/2015/374579

**Published:** 2015-02-19

**Authors:** Valentina Boccia, Alfredo Renga, Giancarlo Rufino, Marco D'Errico, Antonio Moccia, Cesare Aragno, Simona Zoffoli

**Affiliations:** ^1^Department of Industrial Engineering, University of Naples “Federico II,” Piazzale Tecchio 80, 80125 Naples, Italy; ^2^Department of Industrial and Information Engineering, Second University of Naples, Via Roma 29, 81031 Aversa, Italy; ^3^Kell S.r.l., Via E.Q. Visconti 8, 00198 Rome, Italy; ^4^Italian Space Agency, Via del Politecnico, 00133 Rome, Italy

## Abstract

Long gravity waves or swell dominating the sea surface is known to be very useful to estimate seabed morphology in coastal areas. The paper reviews the main phenomena related to swell waves propagation that allow seabed morphology to be sensed. The linear dispersion is analysed and an error budget model is developed to assess the achievable depth accuracy when Synthetic Aperture Radar (SAR) data are used. The relevant issues and potentials of swell-based bathymetry by SAR are identified and discussed. This technique is of particular interest for characteristic regions of the Mediterranean Sea, such as in gulfs and relatively close areas, where traditional SAR-based bathymetric techniques, relying on strong tidal currents, are of limited practical utility.

## 1. Introduction

Study and development of new techniques for monitoring and control of coastal regions are a crucial aspect at the international level [1]. Both large cargo ships and small sailing boats are abundant in coastal areas together with a large number of human activities like fishing, pipeline management, marine research, cable laying, and recreation. Accurate measurements of sea water depth and seabed morphology are, therefore, essential for proper resource management [2]. Acquisition of bathymetric information and production of the relevant digital elevation models (DEMs) of coastal areas can guarantee safe navigation and allow important activities to be conducted such as execution of warp analysis, monitoring of geomorphological risk, and forecasting of potential flooding effects. In addition, accurate measurements of the sea floor morphology can support monitoring of marine pollution and archeological researches in underwater coastal area.

Conventional techniques to perform hydrographic surveys and to obtain bathymetric data are based on scanning the region of interest with echo-sounders [3]. Survey vessels are used to cover the region by planned transects and a dense distribution of bathymetric data is thus retrieved. Different scales of accuracy can be obtained by using different types of echo-sounders and the adoption of inertial sensors with integration of differential GPS allows subdecimeter accuracy to be achieved over shallow waters [4]. Unfortunately, despite the high level of accuracy that it can provide, conventional hydrographic surveying is expensive and complex to realize due to the necessity to implement dedicated* in situ* acquisition campaigns [5].

Several remote sensing methods, such as the use of Light Detection and Ranging (LIDAR) technology [6, 7] or passive optical bathymetry [8–10], have been suggested as potential alternatives or useful support to conventional bathymetric techniques. Indeed, remote sensing methods provide quick coverage of large areas at relatively low cost, thus allowing local depth variations and temporal morphodynamical development of seabed in coastal regions to be potentially monitored [2]. However, those technologies work only with clear water and this represents a significant limit for their applicability [11].

Since the first spaceborne Synthetic Aperture Radar (SAR) was sent into orbit onboard the NASA's SEASAT satellite in 1978 [12], the possibility to retrieve bathymetric data by analyzing the information on the ocean surface provided by SAR has been investigated [13, 14]. SAR imaging has the potential for improved temporal resolution with respect to traditional bathymetric surveys. Revisit time of several days is typically achieved by spaceborne SARs thus allowing regular monitoring even on long time scales. However, due to the inability of SAR signals to penetrate sea water and reach seabed [15], SAR returns a high-resolution 2D image of sea surface. Therefore, bathymetric measurements from SAR data rely on indirect processes, with the sea floor morphology sensed through the effects it produces on sea surface. Since the magnitude of these effects decreases with the local depth, SAR-based bathymetry cannot be applied offshore and over deep water areas. Nonetheless, bathymetric coverage of shallow waters and coastal regions can be performed.

The first model explaining how to detect underwater morphology from sea surface features using SAR data has been proposed by Alpers and Hennings [16]. The model focuses on the variation of strong currents over sand banks. Due to conservation of mass, when a current flows perpendicular to a sand bank its speed increases over the ridge and decreases after the ridge. These variations in current speed modify sea surface roughness thus affecting radar backscatter over that area [17–19]. From then on, several researches have been conducted to estimate seabed morphology from SAR data through this current-based bathymetric theory [5, 13, 14].

A different approach for SAR-based bathymetric data retrieval has been recently applied to high-resolution SAR data [20]. It relies on the variations in wave propagation direction and wavelength that occur when swell waves pass from deep to shallow water [21]. When swell waves propagate on sea surface, refraction and shoaling phenomena can be tracked in SAR images from offshore to the shoreline [22]. By using a proper modelling to relate these changes in swell wave characteristics to the sea water depth [21], bathymetric data can be retrieved [23].

The present paper focuses on SAR-based bathymetry exploiting swell wave propagation. This is a promising technique for characteristic regions of the Mediterranean Sea, such as in gulfs and relatively close areas, where currents are extremely weak and cannot be used to infer bathymetry. Based on the consideration that the linear dispersion relation is generally adopted for swell-based bathymetry, the paper develops an error budget model for this dispersion relation. Moreover, the paper discusses viability and potential performance for the case in which SAR imaging is used to provide the linear dispersion relation with suitable input data. According to authors' knowledge of existing literature, limited discussions on those topics are available [20, 23, 24].

The paper is organized as follows.  Section 2 describes the main phenomena related to swell waves propagation that allow seabed morphology to be detected in coastal areas from SAR images and capability of SAR to image them.  Section 3 investigates applicability of the linear dispersion relation and provides some guidelines for its proper exploitation on SAR images for bathymetric applications.  Section 4 describes the performed error budget analysis for depth retrieval sensitivity to swell parameters when linear dispersion relation is applied with specific reference to SAR-based bathymetry.  Section 5 reports a discussion on the main outcomes derived through the paper.

## 2. Bathymetric Features on Sea Surface and SAR Imagery

Sea surface is usually a combination of many wave components having significant variability in height, frequency, and wavenumber as well as in direction of propagation. Three main kinds of waves can be identified: capillary waves, swell waves, and wind waves. Swell waves are long-period waves with long individual crests which travel far from their region of origin and which tend to be uniform in height, period, and direction of propagation [25–27]. Gravity is their controlling force and causes their propagation once the sea surface is displaced from its rest position. When swell waves travel from deep to shallow water they start feeling the influence of seabed morphology [25].

### 2.1. Bathymetric Sea Surface Features

Effects of seabed morphology on swell waves can be essentially related to two main phenomena which modify swell waves propagation on sea surface. When swell waves approach shallow water areas, their speed and wavelength decrease thus causing an increment in their height due to conservation of energy. This phenomenon is called shoaling. Moreover, if the traveling swell crests are not aligned with contour lines of sea bottom morphology, different portions along the crest exhibit a different magnitude of the shoaling phenomenon. Variation of depth along a wave crest causes different portions of the crest to travel at different speeds, with those parts in shallower water being more decelerated than those in deeper water. The overall result is a change of the swell travel direction to realign their crests and become parallel to the shoreline. This process is called refraction and it takes place until the swell crests become parallel to the shoreline or the wave breaks [20, 28]. This implies that rays, that is, the virtual lines perpendicular to the wave crests that contain a constant energy flux, converge on local shoals and shallow water areas. Phenomena of swell wave shoaling and refraction due to underwater morphology start appearing in intermediate water depth because surface waves begin to feel the bottom when sea water depth, *h*, is shorter than about half of the swell wavelength, *L* [20]:

(1) $$\begin{array}{c} h<\frac{1}{2}L. \end{array}$$

This means that bathymetry through swell wave modulation can be performed only for water depth values lower than half the swell wavelength, that is, the limit water depth value. This limit cannot be univocally defined since swell wavelength depends on the considered case.

### 2.2. SAR Imaging of Bathymetric Sea Surface Features

Microwave signals emitted by SAR are able to penetrate into sea water just a few centimetres [15] and, therefore, they are unable to reach the seabed [13]. This means that the echoed signals received from sea can be considered as sea surface echoes measuring the surface roughness encountered by the transmitted microwave signals. Specifically, the backscatter patters in SAR images of sea surface are due to the presence of wind-generated short waves, called Bragg waves [13, 25, 29]. These are waves that satisfy the Bragg condition thus generating constructive interference in the direction of the SAR sensor. In the case of total lack of wind, sea surface is smooth and it reflects the SAR signal aside [25] generating no echo to the sensor antenna.

When swell waves fully characterize sea surface at the moment of SAR image acquisition, they can be imaged as amplitude modulations of the radar echoes because they modulate the sea surface roughness distribution [13, 25]. This mechanism is related to the following three mechanisms: hydrodynamic modulation, tilt modulation, and velocity bunching modulation [22]. On the contrary, when other kinds of waves, for example, wind sea [24], significantly affect sea surface parameters, a proper visualization of swell waves may be prevented.

In general, the imaging mechanism of sea surface by SAR is not linear. As a consequence, a SAR image cannot be interpreted as a picture of the surface. Complex models are necessary to retrieve sea spectrum information (e.g., see [30, 31]). However specific conditions exist in which linear imaging can be assumed [31]. This is the case of not extreme wind speed and sea state, absence of currents, and swell patterns characterized by wavelengths that are sufficiently far from the cut-off conditions [20]. Experimental results demonstrate that when these conditions are satisfied, the swell wavelengths imaged by SAR represent an unbiased estimate of the true swell wavelengths [24].

Particular attention should be given to surface currents since they can cause variations in swell phase velocity and wavelength which generate the same effects as seabed morphology in shallow water [21, 22, 24, 25]. This means that SAR images are useful to investigate seabed morphology only if acquired under weak current velocity. Quantitatively, current velocity larger than 0.05 m/s should be avoided [24]. Moreover, values of the incidence angle of SAR images should be as short as possible thus increasing the backscattering from sea surface and reducing undesirable smearing effects [13].

To properly identify bathymetric sea surface features, SAR images with swell waves propagating along the flight direction should be avoided since the velocity bunching phenomenon may cause the minimum discernible wavelength to be longer than that of swell waves propagating along the ground-range direction [13, 22, 25]. Equation (2) shows an empiric expression [20] which links the minimum detectable value of wavelength for swell waves travelling along the azimuth direction, *L*_*Min*⁡∣Azim_, to the slant range, *R*, platform velocity, *V*, and significant wave height, *H*_*s*_:

(2) $$\begin{array}{c} L_{\mathrm{Min}\;|\;\text{Azim}}=\frac{R}{V}\sqrt{H_{s}}. \end{array}$$

On the contrary, SAR capability to detect swell waves traveling along the ground-range direction strictly depends on the ground-range resolution of the radar system. In detail, ground-range resolutions lower than five times the swell wavelength allow accurate and reliable measurements of the swell wavelength to be performed.

For swell travelling along a generic direction, the minimum detectable length ranges from the one along the ground-range dimension, *L*_*Min*⁡∣Range_, to that along the azimuth direction, *L*_*Min*⁡∣Azim_. To a first order, being *ϕ* the angle between the direction of propagation of the swell waves and the platform flight direction, the value of the minimum detectable wavelength, *L*_*Min*⁡_, can be estimated as

(3) LMin⁡=LMin⁡ ∣ Range21−cos⁡2ϕ+LMin⁡ ∣ Azim21−cos⁡2ϕ+π.

Equation (3) matches the results presented in [32].

**Figure 1:**
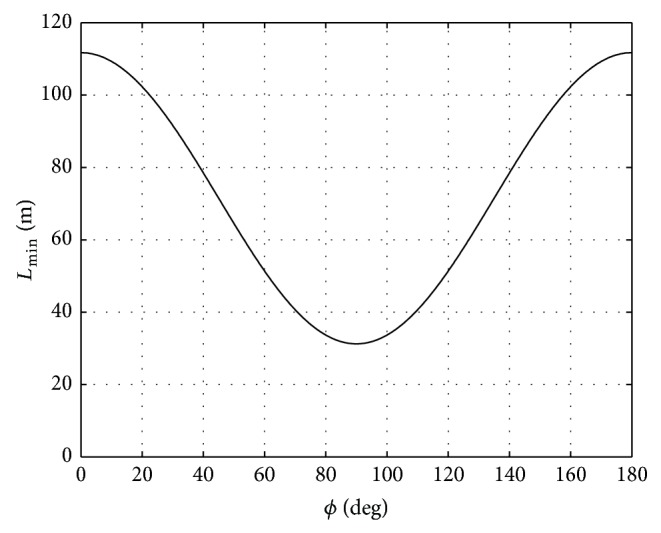
Simulated trend of the minimum value for detectable swell wavelength obtained from (3) by using typical values for ALOS PALSAR working in stripmap mode (6.25 m ground-range pixel spacing); that is, *L*_*Min*⁡∣Range_ = 31.25 m, *V* = 7.59 km/s, *R* = 663 km, and for *H*_*s*_ = 1 m.

Figure 1 depicts the simulated trend of the minimum detectable swell wavelength obtained by using typical values for ALOS (Advanced Land Observing Satellite) PALSAR working in stripmap mode, that is, 6.25 m ground-range pixel spacing, *L*_*Min*⁡∣Range_ five times larger than the resolution, that is, 31.25 m, 7.59 km/s for the satellite velocity, and 848 km for the slant range (corresponding to about 38° incidence angle), and by considering 1 m for the significant wave height. As expected, the performed simulation shows that the value of the minimum detectable wavelength varies in the range [31.25; 111.73] m, shorter for swell waves propagating along the ground-range direction and longer for swell waves propagating along the azimuth one.

**Figure 2:**
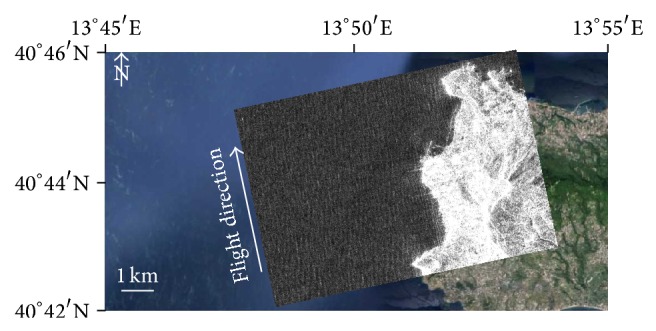
Subset of the ALOS PALSAR stripmap image, 6.25 m × 6.25 m pixel spacing, acquired on February 13, 2007, at 20:28 UTC, covering the west coast of the Ischia Island in the Gulf of Naples, Italy (background image © Google Earth); swell waves propagate along the ground-range direction and phenomena of shoaling and refraction are well visible approaching the coastline.

Figure 2 shows a subset of an ALOS stripmap image, 6.25 m × 6.25 m pixels spacing, covering the west coast of the Ischia Island in the Gulf of Naples, Italy. The sea surface is characterized by a swell field travelling along the ground-range direction with swell wavelength in the range [70 m; 160 m]. This is an example of the conditions in which shoaling and refraction can be tracked and linear SAR imaging can be assumed. Hence, bathymetry can be inferred if a relation is introduced to model swell parameters as a function of water depth. This relation is typically called dispersion relation as discussed in the next section.

## 3. Linear Dispersion Relation for SAR-Based Bathymetry

The dispersion relation models the hydrodynamic phenomenon of swell waves modulation due to water depth variations by considering the governing physical processes, particularly the restoring force. Several mathematical expressions, characterized by different levels of details, exist to describe the relation between swell wave parameters and water depth [21, 26, 32–35]. Extensive studies have been performed on the applicability of those expressions for SAR-based bathymetric data retrieval. Results have shown that nonlinear theories require values of additional swell wave parameters to be known, for example, swell wave height and period, which cannot be directly calculated by using SAR [32, 33] or, at most, they can be retrieved with high level of uncertainty. Such uncertainties can be so large that the resulting uncertainty in the water depth is larger than the one obtained by using the linear dispersion relation, that is, by neglecting the effects of nonlinear processes. Therefore, the finite depth linear version of the dispersion relation for swell waves has been universally recognized as the most suitable for SAR-based bathymetric data retrieval [20, 23, 24, 36].

### 3.1. Linear Dispersion Relation

The linear dispersion relation is based on the Airy wave theory, that is, the linear wave theory. When the assumption of small amplitude waves is made, the mechanism of swell waves propagation on the surface of a homogeneous fluid layer can be represented by a linear theory. This allows a sinusoidal wave profile [25, 37] to be assumed without losing generality. Indeed, the whole reasoning can be applied also to more complex wave profiles since they can be modelled as the superposition of sinusoidal waves with different wavelengths.

The standard expression of the linear dispersion relation in intermediate water depth, that is, for water depth in the range [(1/20)*L*; (1/2)*L*], relates the swell wave angular frequency *ω*, that is, the frequency at which the swell waves oscillate as measured by an observer fixed in the medium, the gravity acceleration *g*, water depth *h*, and swell wavenumber *k* [25–27, 37, 38]:

(4) $$\begin{array}{c} \omega^{2}=gk\tanh\left(kh\right). \end{array}$$

By considering the relationship between swell wavenumber and swell wavelength, that is, *k* = 2*π*/*L*, and the one between swell frequency and swell period, that is, *ω* = 2*π*/*T*, a more useful form of the linear dispersion relation for SAR-based bathymetry data retrieval in intermediate water depth can be obtained:

(5) $$\begin{array}{c} h=\frac{L}{2\pi }\tanh^{-1}\left(\frac{2\pi L}{T^{2}g}\right). \end{array}$$

The phase speed of swell waves indicates the speed at which a wave crest moves and is given by

(6) $$\begin{array}{c} c=\frac{\omega }{k}. \end{array}$$

The phase speed is a function of depth, swell period, and wavelength. This is the main reason explaining why bathymetry can be performed in intermediate water. Differently, mathematical expressions of the linear dispersion relation both in deep water and shallow water are demonstrated to be not useful to estimate bathymetry [34, 35]. Indeed, the linear dispersion relation in deep water (*h* > (1/2)*L*) is

(7) $$\begin{array}{c} \omega =\sqrt{gk} \end{array}$$

and the phase speed of swell waves [26] is

(8) $$\begin{array}{c} c=\sqrt{\frac{g}{k}}=\frac{gT}{2\pi }. \end{array}$$

Equation (8) shows that the phase speed in deep water is only dependent on the swell period thus causing longer, lower-frequency waves to propagate faster than the shorter, higher-frequency waves and leading to dispersion of the wave components, which does not depend on the wavelength.

Similarly, the shallow water (*h* < (1/20)*L*) dispersion relation is

(9) $$\begin{array}{c} \omega =k\sqrt{gh} \end{array}$$

and the phase speed of swell waves is

(10) $$\begin{array}{c} c=\sqrt{gh} \end{array}$$

thus depending only on water depth. This means that all waves travel at the same speed independently of their wavelength or, equivalently, that there is no relation between wavelength and depth.

### 3.2. Applicability of the Linear Dispersion Relation

This section analyzes the theoretical behavior of the linear dispersion relation in intermediate water depth. Therefore, for each considered swell wavelength only the values of water depth less than half that wavelength will be considered. In addition, only the wavelength satisfying the relation |2*πL*/*T* ^2^ *g*| < 1 has to be taken into account due to the tanh^−1^ function (see (4)). This means that, for each swell wavelength, a lower limit value for the swell period $T_{\text{MIN}}=\sqrt{2\pi L/g}$ has to be considered.

**Figure 3:**
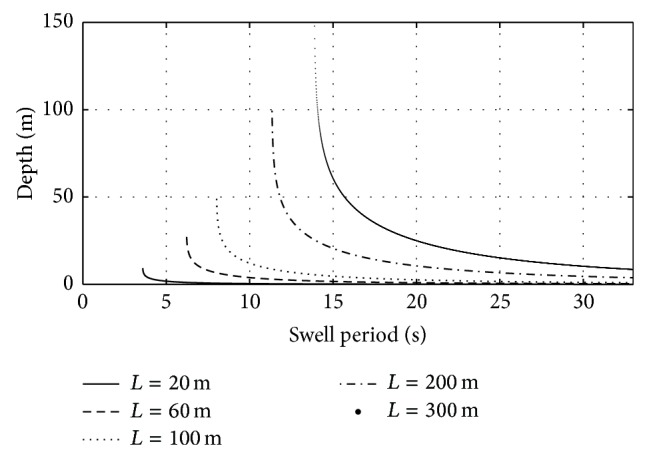
Sea water depth as function of swell period for different values of swell wavelength, *L*, in the range [20; 300] m, according to the linear dispersion relation starting from intermediate water depth up to shoreline.

**Figure 4:**
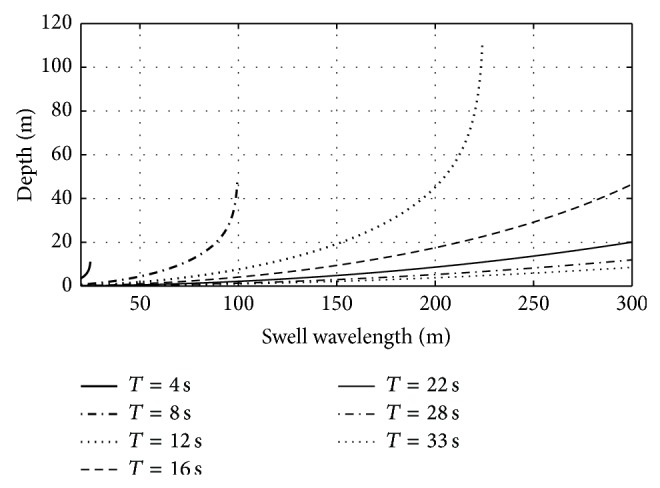
Sea water depth as function of swell period for different values of swell period, *T*, in the range [4; 34] s, according to the linear dispersion relation starting from intermediate water depth up to shoreline.

Figures 3 and 4 show the relation between water depth, swell wavelength, and swell period in coastal areas for typical ranges of swell wavelength *L* ∈ [20; 300] m, and swell period *T* ∈ [4; 33] s.  Figure 3 shows that, for constant swell wavelength, the water depth decreases when the swell period increases. On the contrary, if swell period is constant, swell wavelength decreases when water depth decreases too; the longer the swell wavelength is, the steeper the slope of this trend is. The absolute value of the slope increases at short swell wavelength thus leading to large uncertainty in water depth estimation based on swell period. Indeed, a small error in the swell period is amplified by the linear dispersion relation for short values of the swell wavelength thus leading to a large error in the estimated water depth.

Figure 4 shows that the longest acceptable value for the measured swell wavelength, that is, the largest acceptable value for the estimated water depth, depends on the considered value of swell period. The shorter the swell period is, the shorter the maximum acceptable swell wavelength is. In particular, for large swell periods this value is outside the range of valid swell wavelength. Absolute value of the slope increases for long swell wavelength thus leading to large uncertainty in water depth estimation especially for little swell period. Indeed, even a small error in swell wavelength leads to a large error in the estimated water depth.

According to the performed analysis a further remark can be made. When the swell period is sufficiently large and the local depth is quite limited, variations in the swell period are expected to generate very limited variations in the estimated water depth; that is, the sensitivity of water to swell period is very low.

This property turns to be very useful for SAR-based bathymetry since it allows a first-guess approach to be used to estimate swell period (see Section 3.3).

### 3.3. Guidelines for the Exploitation of the Linear Dispersion Relation on SAR Images for Bathymetric Applications

When swell waves fully characterize sea surface and linear imaging can be assumed, SAR images of coastal areas can be used to indirectly infer seabed morphology from shoaling and refraction phenomena [39]. The maximum detectable sea water depth can be estimated by considering the specific range of swell wavelength present on the SAR image. Imaged swell wavelengths can be calculated from SAR images by using spectral analysis [20, 22–25, 35, 36, 39]. The two-dimensional spectrum of a small region of the SAR image has to be retrieved in order to convert it into the directional wave spectrum of the local wave field. The peak in the two-dimensional spectrum indicates the dominant wavelength and the dominant wave direction of all the waves visible in the subimage [20]. Concerning the period, it cannot be measured by the aforementioned spectrum analysis. Other sources, for example, buoys or weather services, could be used. Alternatively, a first-guess approach [20, 40] can be implemented by estimating the swell period with a first guess for water depth from an available low-resolution topography dataset. Thanks to the linear dispersion relation and to the measured swell wavelength from SAR image, the value of the swell period can be estimated. Following the properties of the linear dispersion relation, the swell period calculated offshore, if sufficiently long, can be used for the whole swell ray up to the shoreline, generating very low errors in the estimation of water depth. Moreover, it would be advisable to retrieve the swell period in correspondence of a sea surface area as close as possible to the beginning of the intermediate water depth domain so as to reduce errors in the retrieved water depth. Once values of swell wavelength and period have been estimated, (5) can be applied to retrieve the water depth corresponding to the considered subimage [20]. To analyze a larger portion of the SAR image, the spectral analysis has to be conducted by scanning the area of interest with small windows. A raster approach can be used by considering scanning windows at a predetermined distance [36]. The Fast Fourier Transform (FFT) is applied for each location of the window; a two-dimensional image spectrum is retrieved and converted into the directional wave spectrum of the wave field. This process is performed until the corner points of the scanning window reach the shoreline. Finally, it is worth noting that the capability to infer seabed morphology is strictly related to the values of swell wavelength. Hence, it is expected that only sea bottom features characterized by a scale length that is at least of the same order of magnitude as the local peak wavelength of the swell waves can be characterized [39].

## 4. Error Budget Analysis of Linear Dispersion Relation and Application to SAR-Based Bathymetry

An error budget model is presented in this section to investigate the potential performance of the linear dispersion relation. For any given model of water depth estimation, *h* = *h*(*S*_1_, *S*_2_,…, *S*_*n*_), the basic propagation formula for variance of water depth, *σ*_*h*_^2^, in terms of all the related error sources (*S*_1_, *S*_2_,…, *S*_*n*_) is given by

(11) σh2=∑i∂h∂Si2σSi2+∑i,ji≠j∂h∂Si∂h∂SjσSi,Sj,i=1,…,n; j=1,…,n.

With reference to the linear dispersion relation (5), the statistical correlation between error sources, that is, the second summation in (11), is neglected. The variance of the estimated water depth is obtained by adding *σ*_*h*|_*L*__^2^, that is, the square of the water depth uncertainty due to the one in the swell wavelength, *σ*_*L*_, to *σ*_*h*|_*T*__^2^, that is, the square of the water depth uncertainty due to the one in the swell period, *σ*_*T*_:

(12) $$\begin{array}{c} \sigma_{h}^{2}={\left(\frac{\partial h}{\partial L}\right)}^{2}\sigma_{L}^{2}+{\left(\frac{\partial h}{\partial T}\right)}^{2}\sigma_{T}^{2}=\sigma_{{\left.h\right|}_{L}}^{2}+\sigma_{{\left.h\right|}_{T}}^{2}. \end{array}$$

The analytic formula for the sensitivities, that is, the partial derivatives ∂*h*/∂*L* and ∂*h*/∂*T*, can be derived by differentiation of the linear dispersion relation (5):

(13) $$\begin{array}{c} \frac{\partial h}{\partial L}=\frac{1}{2\pi }\tanh^{-1}\left(\frac{2\pi L}{T^{2}g}\right)+\frac{L}{T^{2}g}\frac{1}{1-{\left(2\pi L/T^{2}g\right)}^{2}}, \end{array}$$

(14) $$\begin{array}{c} \frac{\partial h}{\partial T}=-L\frac{1}{1-{\left(2\pi L/T^{2}g\right)}^{2}}\frac{2L}{T^{3}g}. \end{array}$$

Equations (13) and (14) show that *σ*_*h*|_*L*__ and *σ*_*h*|_*T*__ depend on wavelength and period of the swell wave field propagating in the considered portion of sea surface. In particular, the larger the swell period is, the lower the sensitivities are. On the contrary, if a short value of swell period is assumed, large sensitivities occur thus leading to a large *σ*_*h*_^2^ also with limited *σ*_*L*_ and *σ*_*T*_.

In order to avoid large errors in the estimation of water depth, an upper limit for sensitivities is introduced. Ranges of acceptable values should, therefore, be defined for swell wavelength and swell period, respectively, so that only values of *L* and *T* which give

(15) $$\begin{array}{c} \left|\frac{\partial h}{\partial L}\right|\leq {\left|\frac{\partial h}{\partial L}\right|}_{\text{limit}}, \end{array}$$

(16) $$\begin{array}{c} \left|\frac{\partial h}{\partial T}\right|\leq {\left|\frac{\partial h}{\partial T}\right|}_{\text{limit}} \end{array}$$

will be used for bathymetric data retrieval. Limit values for sensitivities must be selected depending on the accuracy desired for the bathymetric survey.

**Figure 5:**
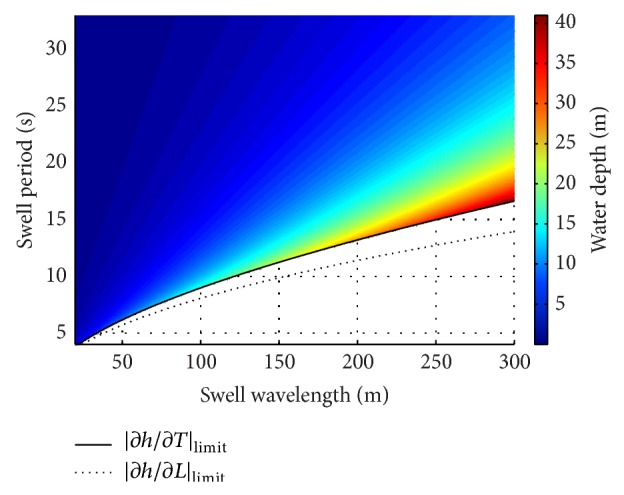
Swell wavelengths and periods which provide |∂*h*/∂*L*| = |∂*h*/∂*L*|_limit_ (dotted line) and |∂*h*/∂*T*| = |∂*h*/∂*T*|_limit_ (continuous line) together with the water depths calculated from the linear dispersion relation in the considered admissible area of the (*L*, *T*) plane.

In the present paper, the cut-off value of 7.76 has been considered reasonable for both |∂*h*/∂*L*|_limit_ and |∂*h*/∂*T*|_limit_. It is equivalent to the value of |∂*h*/∂*T*| obtained from (14) by considering *L* = 200 m and *h* = 30 m.  Figure 5 shows that for each value of swell wavelength, two lower limit values of swell period are obtained: one related to |∂*h*/∂*L*|_limit_ and the other one related to |∂*h*/∂*T*|_limit_. Since (15) and (16) must be satisfied at the same time, only values (*L*, *T*) in the area above both the two curves can be used. In the considered case, the admissible area is the same as considering only condition (16). The minimum acceptable swell period rises as the swell wavelength increases. The more the two curves are close to each other, the more the values of |∂*h*/∂*L*| on the boundary of the admissible area are close to |∂*h*/∂*L*|_limit_.

Figure 5 also shows that setting the upper limit for sensitivities is the same as considering a range of potentially inferable values of water depth. As expected, values of water depth rapidly increase approaching the |∂*h*/∂*T*|_limit_ curve but, in the considered case, values within 30 m are obtained in almost the whole admissible area of the (*L*, *T*) plane. The above-reported considerations are of general validity; that is, they depend only on the properties of the dispersion relation and so they hold independently of the technique used to calculate wavelength and period. On the contrary, uncertainties in the error sources, that is, in swell wavelength, *σ*_*L*_, and in swell period, *σ*_*T*_, depend on the adopted technique.

With specific reference to SAR imaging, the value of *σ*_*L*_ basically depends on the spatial resolution of the inspected SAR image. On the other hand, the value of *σ*_*T*_ depends on the accuracy of the sources that provide the swell period or, in case a first guess approach is used, on the accuracy of the assumed offshore water depth. Ranges of possible values for *σ*_*L*_ and *σ*_*T*_ have been considered to evaluate the performance of the linear dispersion relation for bathymetric data retrieval. Typical values of high spatial resolution (spotlight SAR image) and medium spatial resolution (stripmap SAR image) have been adopted as minimum and maximum values for *σ*_*L*_, respectively. Therefore, values of *σ*_*L*_ ∈ [2; 10] m have been considered in this work [41, 42]. On the other hand, the minimum and maximum values for *σ*_*T*_ have been retrieved from (12) by assuming *σ*_*h*|_*T*__ = 1 m and *σ*_*h*|_*T*__ = 10 m, respectively, with |∂*h*/∂*T*| = |∂*h*/∂*T*|_limit_ = 7.76 m/s. Therefore, values of *σ*_*T*_ ∈ [0.129; 1.29] s have been considered.

**Figure 6:**
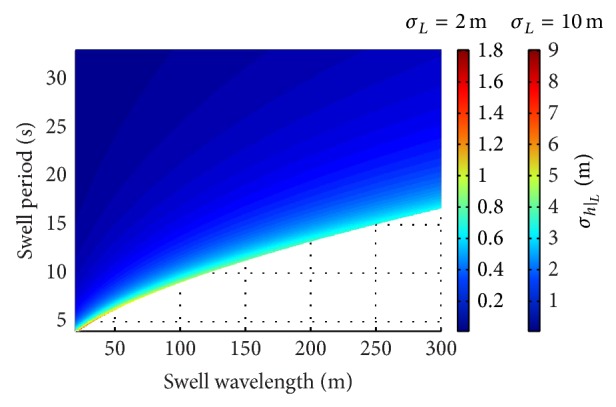
Uncertainty in the estimated water depth due to that in swell wavelength in the considered admissible (*L*, *T*) area for *σ*_*L*_ = 2 m and *σ*_*L*_ = 10 m.

Figure 6 shows the resulting values of *σ*_*h*|_*L*__ for (*L*, *T*) in the considered admissible ranges by assuming the minimum and the maximum values of uncertainty in swell wavelength, respectively. A large zone in the (*L*, *T*) plane is characterized by low values of *σ*_*h*|_*L*__ up to about 0.5 m for *σ*_*L*_ = 2 m and to about 2.3 m for *σ*_*L*_ = 10 m. Values of *σ*_*h*|_*L*__ rise in a small region close to the boundary. For long swell wavelength, *σ*_*h*|_*L*__ rises up to 0.7 m for *σ*_*L*_ = 2 m and to about 3.5 m for *σ*_*L*_ = 10 m. For low *L* and *T*, instead, *σ*_*h*|_*L*__ can reach values up to 1.8 m for *σ*_*L*_ = 2 m and to 9 m for *σ*_*L*_ = 10 m since values of |∂*h*/∂*L*| are close to |∂*h*/∂*L*|_limit_ in that region.

**Figure 7:**
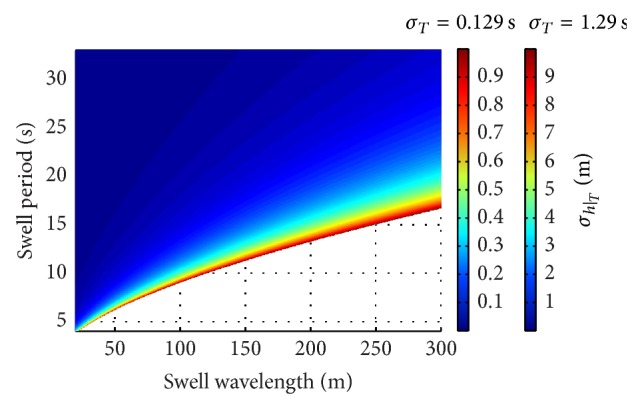
Uncertainty in the estimated water depth due to that in the swell period in the considered admissible (*L*, *T*) area for *σ*_*T*_ = 0.129 s and *σ*_*T*_ = 1.29 s.

Similarly, Figure 7 shows the calculated values of *σ*_*h*|_*T*__ for (*L*, *T*) in the considered admissible ranges by assuming the minimum and the maximum values of uncertainty in swell period. As expected, the values of *σ*_*h*|_*T*__ are quite low for large swell period. A large zone in the (*L*, *T*) plane is characterized by short values of *σ*_*h*|_*L*__ up to about 0.4 m for *σ*_*T*_ = 0.129 s and up to about 4 m for *σ*_*T*_ = 1.29 s. They rapidly increase approaching the boundary and achieve values up to 1 m for *σ*_*T*_ = 0.129 s and up to 10 m for *σ*_*T*_ = 1.29 s. The values of *σ*_*h*|_*T*__ increase more steeply than *σ*_*h*|_*L*__ in the area next to the boundary. This is because |∂*h*/∂*T*| quickly rises in that region up to the limit value |∂*h*/∂*T*|_limit_.

**Figure 8:**
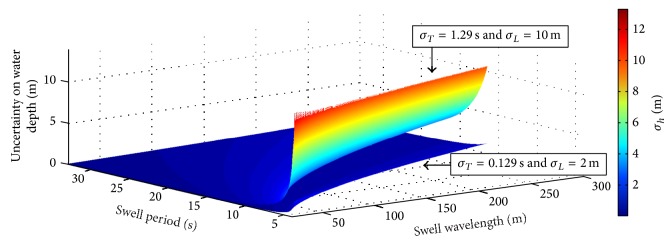
Uncertainty in the estimated water depth in the considered admissible (*L*, *T*) area for the minimum and maximum values for both *σ*_*T*_ and *σ*_*L*_.

Finally, the overall uncertainty in water depth has been calculated (12).  Figure 8 shows the obtained values of *σ*_*h*_ in the two limit cases for both *σ*_*T*_ and *σ*_*L*_. When the minimum values are considered, that is, *σ*_*T*_ = 0.129 s and *σ*_*L*_ = 2 m, the uncertainty in water depth is estimated to be not larger than about 0.6 m for almost all the acceptable (*L*, *T*) values. It increases in the region next to the boundary and, for short values of swell wavelength and period, it reaches values up to 2 m. On the other hand, for maximum input uncertainties, that is, *σ*_*T*_ = 1.29 s and *σ*_*L*_ = 10 m, *σ*_*h*_ gets up to about 4.6 m for a large area in the (*L*, *T*) plane and rises steeply up to about 13 m next to the boundary.

## 5. Discussion

The linear dispersion relation has been considered for swell-based bathymetry. Its viability and potential performance for water depth retrieval from SAR images of sea surface have been investigated. This is, in general, not trivial since it is related to several issues.

With specific reference to sea surface state, the considered approach is able to infer sea water depth when swell waves fully characterize the sea surface at the moment of the SAR acquisition, that is, when no other significant features are present and linear SAR imaging can be assumed. Those areas can be individuated by a visual inspection of the SAR image since swell waves are imaged by SAR as long, regular, and bright-and-dark alternating stripes (see Figure 2) [39]. Disturbing phenomena, if present, will not significantly affect swell parameters or, alternatively, they must be filtered out by proper preprocessing steps [43].

Under those conditions, the performed analysis has revealed that the values of sea water depth that can be potentially retrieved by using the linear dispersion relation depend on the detected values of swell wavelength, *L*, and period, *T*, as well as on the maximum admissible value of uncertainty. In turn, also the uncertainty in the retrieved sea water depth, *σ*_*h*_, depends on the detected values of swell wavelength and period as well as on their uncertainties. The use of a maximum limit value for sensitivities of water depth to swell parameters has been suggested depending on the desired level of accuracy in sea water depth estimation. In this way, an area of acceptable values in the (*L*, *T*) plane can be defined.  Figure 5 reports the admissible (*L*, *T*) values with typical ranges for swell wavelength (*L* ∈ [20; 300] m) and swell period (*T* ∈ [4; 33] s) of coastal areas by adopting a cut-off value of 7.76 for both |∂*h*/∂*L*|_limit_ and |∂*h*/∂*T*|_limit_. Under the above assumptions, linear dispersion relation is theoretically able to retrieve values of water depth up to about 40 m. The error budget analysis has been performed by using two values, 1 m and 10 m, for the uncertainty in swell wavelength, *σ*_*L*_, typical of high and low spatial resolution of SAR, respectively, and by using two values, 0.129 s and 1.29 s, for the uncertainty in swell period, *σ*_*T*_. Linear imaging of sea surface and short values of incidence angle of SAR images are assumed. The results show that the uncertainty in the estimated water depth due to that in swell wavelength, *σ*_*h*|_*L*__, and the one due to that in swell period, *σ*_*h*|_*T*__, are limited in a large zone in the (*L*, *T*) plane. Figures 6 and 7 show that their values increase in a small region close to the boundary of the admissible area of the (*L*, *T*) plane. Tables 1 and 2 report the resulting values of *σ*_*h*|_*L*__ and *σ*_*h*|_*T*__, respectively, for some (*L*, *T*) values by assuming the minimum and the maximum values of uncertainty in swell parameters.

The performed analysis has shown that the values of the overall uncertainty in water depth, *σ*_*h*_, are lower than 0.6 m for almost all the acceptable (*L*, *T*) region in the case of the minimum considered values of both *σ*_*L*_ and *σ*_*T*_. Similarly, in the case of the maximum considered values of both *σ*_*L*_ and *σ*_*T*_, values of *σ*_*h*_ are within 4.6 m for almost all the acceptable (*L*, *T*) region.  Table 3 reports the resulting values of *σ*_*h*_ for some (*L*, *T*) pairs by assuming the minimum and the maximum values of uncertainty in swell parameters.  Figure 8 and Table 3 show that they increase in the region next to the boundary and, when short values of swell period are used, *σ*_*h*_ reaches its maximum, that is, a value between 2.05 m and 13.2 m (in the case, resp., of the minimum and the maximum considered values for both *σ*_*L*_ and *σ*_*T*_).

Finally, it is important to point out that the swell-based technique is able to generate contour-like charts to describe the bottom morphology. It has been conceived to detect bathymetric features that can be properly represented by a series of contour lines spaced by a quantity at least of the same order of magnitude as the swell peak wavelength in that area. As a consequence, sharp water depth variations are filtered out by the method and cannot be detected. Hence, the bathymetric detection capability of swell-based bathymetry is strictly dominated by the morphological characteristics of seabed, no matter if it is sandy or rocky. It is worth noting that the detection of underwater features can be enhanced by a careful selection of the SAR images to process. Specifically, particular attention should be paid to the visual inspection of SAR images with a swell waves field propagating along the flight direction of the SAR sensor. Indeed, due to the velocity bunching phenomenon, azimuth propagating waves can be smeared and therefore of limited utility, if not characterized by a wavelength significantly longer than the cut-off length of (2). The availability of ancillary information, for example, related to wind speed or presence of surface currents, even if not strictly necessary, can represent a valuable aid in assessing the applicability of the linear SAR imaging model.

## 6. Conclusions

The present paper has reviewed the main phenomena related to swell waves propagation that allow underwater bathymetric features to be detected in coastal areas from SAR images. The properties and features of the generally adopted expression of the dispersion relation for SAR-based bathymetric data retrieval, that is, the finite depth linear version of the dispersion relation for swell waves, have been discussed. Its viability and potential performance when applied to SAR-based bathymetry have been analyzed. An error budget model has been developed to assess capabilities and limits of this approach.

Typical ranges of swell wavelength ([20; 300] m) and swell period ([4; 33] s) in coastal areas have been considered. The performed analysis has shown that values of water depth up to 40 m can be retrieved in these conditions by using the linear dispersion relation. Two typical values of high and low spatial resolution of SAR, respectively, have been adopted for the uncertainty in swell wavelength. Moreover, two values have been used for the uncertainty in swell period. The performed study has shown that the values of uncertainty in the estimated water depth are lower than 0.6 m (4.6 m) for almost all the acceptable values of wavelength and period, in the case of the minimum (maximum) values of uncertainties on swell parameters that were considered. These results suggest that SAR-based bathymetry can be a potential alternative or a useful support to conventional bathymetric techniques. Planned future activities will deal with applying this approach to real data sets and SAR images, including both X-band and L-band SAR data from COSMO-SkyMed and ALOS missions.

## Figures and Tables

**Table 1:** Uncertainty in the estimated water depth due to that in swell wavelength, *σ*_*h*|_*L*__, for different (*L*, *T*) and *σ*_*L*_ values.

*σ*_*L*_ = 2 [m]			*σ*_*L*_ = 10 [m]		
*L* [m]	*T* [s]	*σ*_*h*|_*L*__ [m]	*L* [m]	*T* [s]	*σ*_*h*|_*L*__ [m]
20	33	0.007	20	33	0.03
22.2	4	1.8	22.2	4	9.0
150	13	0.5	150	13	2.3
300	16.6	0.7	300	16.6	3.5
300	33	0.1	300	33	0.5

**Table 2:** Values of uncertainty in the estimated water depth due to that in swell period, *σ*_*h*|_*T*__, for different (*L*, *T*) and *σ*_*T*_ values.

*σ*_*T*_ = 0.129 [s]			*σ*_*T*_ = 1.29 [s]		
*L* [m]	*T* [s]	*σ*_*h*|_*T*__ [m]	*L* [m]	*T* [s]	*σ*_*h*|_*T*__ [m]
20	33	0.0003	20	33	0.003
22.2	4	0.97	22.2	4	9.7
150	13	0.4	150	13	4
300	16.6	1.0	300	16.6	10
300	33	0.067	300	33	0.67

**Table 3:** Values of uncertainty in the estimated water depth, *σ*_*h*_, for different (*L*, *T*) and (*σ*_*L*_, *σ*_*T*_) values.

*σ*_*L*_ = 2 [m] and *σ*_*T*_ = 0.129 [s]			*σ*_*L*_ = 10 [m] and *σ*_*T*_ = 1.29 [s]		
*L* [m]	*T* [s]	*σ*_*h*_ [m]	*L* [m]	*T* [s]	*σ*_*h*_ [m]
20	33	0.008	20	33	0.037
22.2	4	2.05	22.2	4	13.2
150	13	0.6	150	13	4.6
300	16.6	1.22	300	16.6	10.6
300	33	0.133	300	33	0.889
